# Angiosarcoma of the Colon Presenting with Chronic Diarrhea, Rectal Bleeding, and Pelvic Discomfort

**DOI:** 10.7759/cureus.1117

**Published:** 2017-03-26

**Authors:** Asad Jehangir, Brian H Le, Oluwaseun Shogbesan

**Affiliations:** 1 Hospitalist Services, Reading Health System, Reading Hospital; 2 Pathology, Reading Health System, Reading Hospital; 3 Internal Medicine, Reading Health System, Reading Hospital

**Keywords:** angiosarcoma, colorectal, cd31

## Abstract

Colonic angiosarcoma is a rare malignancy representing only 0.0012% of all colorectal malignancies. Due to its non-specific symptoms, diagnosis is often delayed. However, because of the aggressive nature of this malignancy, it is important to keep it in the differential diagnosis of rectal bleeding, pelvic discomfort, chronic diarrhea, and weight loss in patients with risk factors. We present a case of angiosarcoma of the colon in a 74-year-old female with a remote history of chemo-radiation for anal squamous cell carcinoma (ASCC).

## Introduction

Angiosarcomas are rare malignant tumors accounting for less than one percent of the malignant sarcomas [1]. They are generally seen in the skin and soft tissues, and occasionally in the liver, spleen, adrenal glands, ovaries, and breasts [2]. Angiosarcomas of the gastrointestinal tract are uncommon. They are generally seen in the small bowel and usually present with gastrointestinal bleeding and anemia [1, 3]. Colonic involvement is exceedingly uncommon and portends a poor prognosis with widespread metastasis seen in over a third of these patients [2]. Sigmoid colon is the most common site of involvement in the colon (38%) [2]. Diagnosis is often delayed due to non-specific symptoms and pathology mimicking other tumors. Due to the rarity of the tumor, management guidelines are limited.

## Case presentation

**Figure 1:**
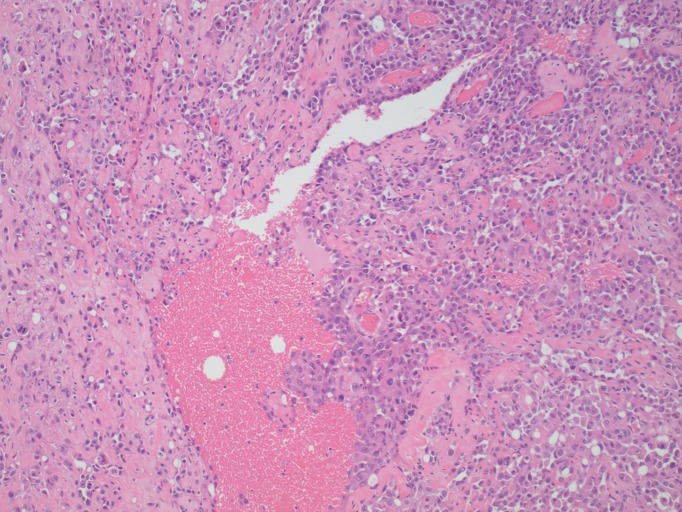
Representative histologic section of tumor, showing infiltrating pleomorphic cells forming vascular channels with blood filled lumina (hematoxylin and eosin stain, 100x original magnification).

**Figure 2:**
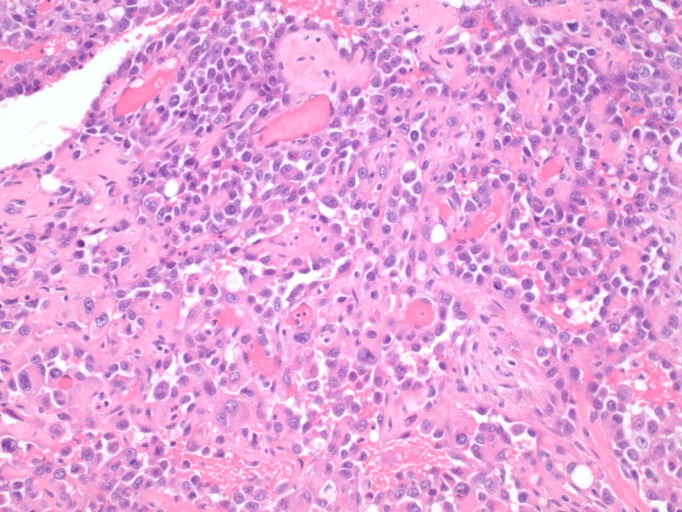
High-power view of the colonic tumor, showing epithelioid morphology (hematoxylin and eosin stain, 200x original magnification).

**Figure 3:**
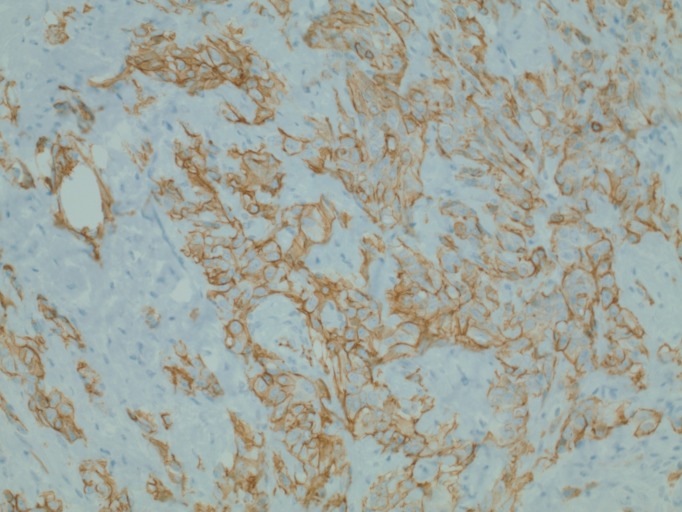
Immunohistochemistry for CD31, a marker associated with vascular endothelial differentiation, shows diffuse reactivity in neoplastic cells (200x original magnification).

A 74-year-old female with a history of anal squamous cell carcinoma (ASCC) (treated with external beam radiation and chemotherapy 11 years prior with an unremarkable surveillance colonoscopy four years previously) and past tobacco use was evaluated for chronic diarrhea of two months' duration. She also complained of rectal bleeding, pelvic cramps, and an unintentional 15-pound weight loss. She denied any nausea or vomiting. She did not report any new medications, sick contacts, changes in diet or recent travel. Her family history was non-contributory. On examination, her vital signs were stable. The abdomen was soft, non-tender, with no lymphadenopathy and a small umbilical hernia. Rectal examination was normal. There were mild external skin changes consistent with prior radiation therapy. Laboratory tests showed mild anemia with a hemoglobin of 11.2 g/dL (reference range 12.0-16.0 g/dL). She underwent a colonoscopy that revealed a stricture of the sigmoid colon, concerning for malignancy versus extrinsic compression. A computerized tomography (CT) of the chest, abdomen, and pelvis showed a thickened area of the sigmoid colon. Two hypodense areas were noted in the right and left lobes of the liver, in addition to infiltrates in the right middle and left lower lung lobes concerning for metastases. An omental implant concerning for metastatic spread was seen adjacent to the cecum. On positron emission tomography (PET) scan, the sigmoid colonic thickening was markedly hypermetabolic. Carcinoembryonic antigen was 0.4 ng/ml (reference range 0.0-3.0 ng/ml) and cancer antigen 125 (CA 125) was also within normal limits at 18 U/ml (reference range 0-21 U/ml). A laparoscopic sigmoid resection with primary anastomosis was performed. On histologic examination, the sigmoid colonic tumor was composed of infiltrative, pleomorphic cells forming vascular channels (Figure 1). On higher magnification, neoplastic cells showed epithelioid morphology (Figure 2). Immunohistochemistry for cluster of differentiation 31 (CD31), a vascular endothelial marker, showed diffuse reactivity (Figure 3), while pan-cytokeratin, a marker of epithelial differentiation, was negative. The morphologic features, in correlation with immunophenotype, were diagnostic of angiosarcoma. Nodal metastases were observed. The overall morphological features and immunophenotype of the tumor were diagnostic of angiosarcoma. The patient subsequently underwent chemotherapy with gemcitabine and docetaxel. However, due to the extent and rapid progression of the disease, she was transitioned to hospice care and later passed away.

## Discussion

Colonic angiosarcoma is a rare malignancy with only 30 cases (24 primary and six metastatic) reported in 2013 [2]. It accounts for 0.0012% of all colorectal malignancies [4]. Risk factors for angiosarcoma include prior chemo-radiation, vinyl chloride, thoratrast, lymphedema, exposure to foreign bodies, and chronic inflammatory conditions (like anorectal ulceration) [3]. The most common presenting symptoms are gastrointestinal bleeding (63%) and perianal pain (54%) [2]. Patients may also have obstructive bowel symptoms (33%) and weight loss (21%), while diarrhea is only seen in 13% of the cases [2]. On examination, an abdominal mass may be palpated [1].

Colonic angiosarcoma commonly metastasizes to the liver, lungs, and bones [2]. Metastases at presentation occur in 38% of the cases [2]. Because of the non-specific symptoms and aggressive nature of these tumors, routine screening colonoscopies may be insufficient for timely assessment, and the diagnosis is delayed in one-fourth of the cases [2,5]. The sigmoid colon is the most frequently involved primary intestinal site [2]. On endoscopy, the mucosa may look normal or show hypervascular mucosa. It may demonstrate an ulcerated or hemorrhagic, protuberant, near-circumferential mass lesion [2, 6]. On microscopy, angiosarcomas may mimic poorly differentiated carcinomas, melanomas and other sarcomas with epithelioid morphology. Of the various immunohistochemical preparations used to aid in diagnosis, CD31, a marker associated with endothelial differentiation, is most often positive (in more than 75% of the cases) [4, 7].

Due to the rarity of the condition, there are no specific guidelines for the diagnosis or the management of colonic angiosarcoma [5]. Surgical resection is offered in most cases with unclear roles of chemotherapy and radiation [5]. Imatinib may be offered to patients who have CD117 positive tumors [2]. Patients with angiosarcoma generally have a poor prognosis because of rapid hematogenous spread [5]. 

## Conclusions

Colonic angiosarcoma is a rare but possible cause of chronic diarrhea, rectal bleeding, and pelvic discomfort in patients with a history of chemo-radiation. Recognition of the angiosarcoma and experience with more cases may help establish management guidelines for patients with this rare but aggressive colonic malignancy. 
